# Thrombosis of abdominal aorta during cisplatin-based chemotherapy of testicular seminoma - a case report

**DOI:** 10.1186/1471-2407-9-459

**Published:** 2009-12-22

**Authors:** Klaus-Peter Dieckmann, Ralf Gehrckens

**Affiliations:** 1 Department of Urology, Albertinen-Krankenhaus, Suentelstrasse 11a, D-22457 Hamburg, Germany; 2 Department of Radiology, Albertinen-Krankenhaus, Suentelstrasse 11a, D-22457 Hamburg, Germany

## Abstract

**Background:**

Vascular complications occurring during cisplatin-based chemotherapy of germ cell tumours are inadequately recognized to date.

**Case Presentation:**

A 49 year old man with advanced seminoma underwent two courses of chemotherapy according to the PEB regimen. Upon restaging, two thrombotic deposits were noted in the descending part of the thoracic aorta and in the infrarenal abdominal aorta, respectively. Although thrombotic plaques caused aortic occlusion of about 30%, no clinical signs of malperfusion of limbs were registered. The patient was placed on anticoagulant therapy. Six months after completion of chemotherapy, thrombotic deposits had completely resolved. In the absence of other predisposing factors, it must be assumed that cisplatin-based chemotherapy represented a strong stimulus for arterial thrombosis in the aorta.

**Conclusions:**

This is the first case of endo-aortic thrombosis during chemotherapy for testicular germ cell cancer. Providers of chemotherapy must be aware of arterial thrombosis even in young patients with testicular cancer.

## Background

Cis-platin based chemotherapy is the cornerstone in the management of metastatic testicular germ cell tumour (TGCT) [1]. The regimen consisting of cisplatin, etoposide and bleomycin (PEB) is usually well tolerated, yet, the toxicity profile of this procedure is significant [2]. Basically, there are acute (immediate) and chronic (late) untoward effects of the PEB protocol. Second malignancies and cardiovascular events constitute well recognized long-term hazards of this chemotherapy [3]. With regard to late cardiovascular problems, cisplatin is thought to initiate degenerative processes of vessel walls, thus causing occlusive vascular disease in the long run. All types of arteries may be involved, and accordingly, there is sound evidence for an excess of myocardial infarctions, arterial hypertension, and cerebral strokes occurring in TGCT patients during long-term follow-up. Only recently it became apparent that cardiovascular complications secondary to cisplatin-based chemotherapy may also occur early during the application of systemic therapy or immediately thereafter [4]. Such complications from cisplatin-based chemotherapy have been encountered in several malignancies [5,6]. However, these events are not well recognized among TGCT patients who are usually younger than most of the patients with other solid malignancies. Thus, we report an unusual case with endo-aortic thrombosis occurring during chemotherapy.

## Case presentation

**Figure 1:**
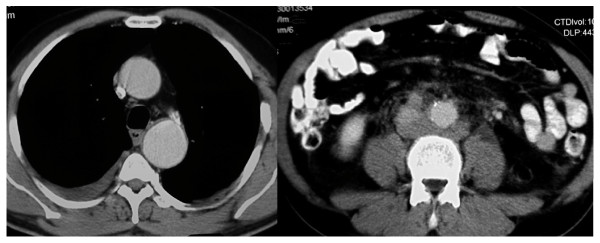
**Contrast enhanced CT-scan at the time before onset of chemotherapy**. 1a: Chest CT. patent aorta, no thrombotic deposits. 2a: abdominal CT: patent aorta, no thrombotic deposits. Minor calcifications in the aortic wall, no major atherosclerotic signs. Note: metastatic deposits around the abdominal aorta; larger metastatic mass located cranial to this section (not shown).

**Figure 2:**
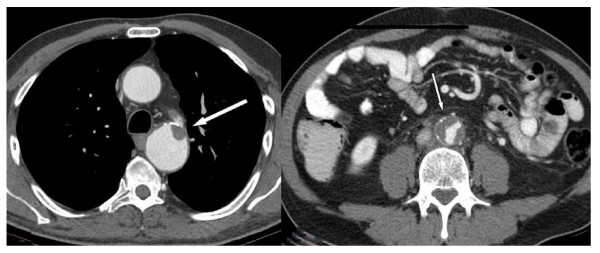
**Contrast enhanced CT-scan after 2 cycles of chemotherapy**. 2a: Chest CT: thrombotic deposit in the descending arch of thoracic aorta (arrow). 2b: abdominal CT: extensive endo-aortic thrombosis (arrow).

**Figure 3:**
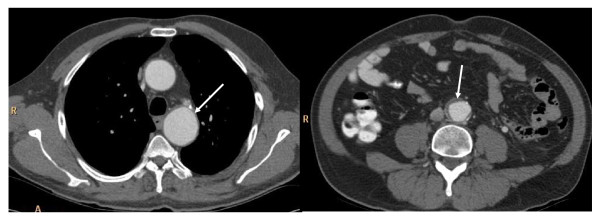
**Restaging CT during follow-up**. Anticoagulant therapy had been applied for 6 months. 3a: Chest CT: complete resolution of thrombus (arrow). 3b: abdominal CT: almost complete regression of endo-aortic thrombosis (arrow). Only minor thickening of aortic wall.

A 49 year old patient presented with advanced seminoma by bulky retroperitoneal disease and left-sided supraclavicular lymphadenopathy, corresponding to stage III (Lugano classification) and to the good prognosis group according to IGCCCG [1], respectively. History involved surgical occlusion of patent Ductus arteriosus Botalli at the age of 17 years. During adult life, no cardiovascular problems had been encountered, clinically. Correspondingly, no major vascular anomaly had been detectable upon staging CT (Fig. 1a, b). Two cycles of chemotherapy according to the PEB regimen were administered in full dose. Clinical course was uneventful. Restaging with abdominal computed tomography (CT) of chest and abdomen revealed regression of metastases and, unexpectedly, endo-aortic thrombotic deposits in the descending arch of the thoracic aorta (Fig. 2a) close to the former junction of aorta and Ductus arteriosus. Surprisingly, another thrombotic deposit was found in the infrarenal abdominal aorta (Fig. 2a, b). As documented in the primary staging CT, these changes had not been present prior to chemotherapy (Fig. 1). Although the abdominal aorta was occluded to approximately 30% by thrombotic material, no significant clinical symptoms were noted by the patient. Anticoagulant treatment with low-molecular heparin and acetylic salicylic acid was instituted. Then, the third cycle of chemotherapy was applied in full dose and without delay. Final restaging after completion of chemotherapy revealed regression of metastases and partial regression of thrombotic deposits at the thoracic and abdominal endo-aortic walls. Anticoagulant therapy was changed to oral warfarin as a permanent medication. Six months later, CT revealed complete resolution of thrombotic material at the thoracic aorta and only minor residual thickening of the abdominal aortic wall (Figs. 3a, b).

## Discussion

Endo-aortic thrombosis is a quite exceptional complication of cisplatin-based chemotherapy. Only few cases have been reported to date involving patients with esophageal carcinoma [7], cervical cancer [8], pancreatic carcinoma and malignant lymphoma [9]. With respect to testicular germ cell tumour, the present case is probably the first event of an endo-aortic thrombosis occurring during chemotherapy. Verdonk et al. reported a celiac trunk thrombosis in a patient undergoing chemotherapy for TGCT [10]. Thrombotic occlusion of peripheral arteries during chemotherapy have been reported in a number of TGCT patients [11-13].

According to Virchow's classical theory, thrombogenesis is principally precipitated by three conditions, first, by compromised blood flow, second, by increased ability of the internal vessel wall to attract blood cells, and third, by systemically increased clotting tendency (humoral factors). Clotting at the aortic wall may arise in the presence of abdominal aortic aneurysm and in extensive atherosclerotic disease, respectively.

Our patient is 49 years old which represents an age category clearly beyond the median age of TGCT patients. As thrombosis is generally associated with increasing age, a slightly increased risk of thrombosis must therefore be considered for this individual, basically. Moreover, he used to be a smoker and there were some minor calcifications detectable in the infrarenal aortic wall upon staging CT. However, there was no clinical sign of systemic atherosclerosis, the body mass index was within normal range (24.8 kg/m^2^), and no other vascular risk factors were revealed upon clinical examination. Thus in all, there was no rationale to consider any compromised aortic blood flow as a pre-existing risk for thrombosis in this patient.

A tempting idea would be to consider the site of the operative occlusion of the Ductus arteriosus Botalli as an area of increased risk of blood cell adhesions. However, this view must remain rather hypothetical because this putative nidus for thrombotic adhesion had been present for more than 30 years without giving rise to blood cell adhesions ever since. Moreover, the critical site at the wall of the thoracic aorta would hardly explain additional downstream thrombotic deposits. Most obviously, chemotherapy constituted a strong stimulus for the forming of arterial thrombosis in our patient. Cisplatin-related hypercoagulability would correspond to condition No. 3 according to Virchow's theory, i.e. increased (systemic) clotting tendency of the blood caused by humoral factors.

The assumption of chemotherapy-related formation of thrombosis is generally in accord with both, findings on the laboratory level e.g. increased levels of von Willebrand factor and other thrombosis-associated factors in patients receiving cisplatinum [14,15] as well as with cisplatin-induced hypomagnesemia and with clinical observations of arterial thromboses and even myocardial infarctions in TGCT patients undergoing chemotherapy [4]. Notwithstanding, the formation of thrombotic plaques in the vessel with highest volume-throughput of the body represents a mostly striking observation.

## Conclusion

Providers of TGCT chemotherapy should be aware of the hazard of arterial thromboses even in large vessels.

## Consent

Written informed consent was obtained from the patient for publication of this case report and any accompanying images. A copy of the written consent is available for review by the Editor-in-Chief of the Journal.

## Competing interests

The authors declare that they have no competing interests.

## Authors' contributions

KPD conceived the study, coordinated the assembling of clinical and radiological findings and wrote the manuscript. RG participated in designing the study, did all radiological analyses and helped to draft the manuscript. All authors have read and approved the final manuscript.

## Pre-publication history

The pre-publication history for this paper can be accessed here:

http://www.biomedcentral.com/1471-2407/9/459/prepub
